# The role of Epstein-Barr virus in NK/T cell lymphoproliferative disorders: molecular mechanisms and potential therapeutic strategies

**DOI:** 10.3389/fimmu.2026.1826581

**Published:** 2026-07-14

**Authors:** Ming Fan, Haiming Kou, Liang V. Tang

**Affiliations:** Institute of Hematology, Union Hospital, Tongji Medical College, Huazhong University of Science and Technology, Wuhan, China

**Keywords:** Epstein-Barr virus (EBV), immune evasion, latent infection, signaling pathways, T/NK cells

## Abstract

Epstein-Barr virus (EBV) is a widely prevalent lymphotropic γ-herpesvirus, with approximately 95% of the population showing evidence of infection at some point during their lifetime. While most infections are asymptomatic or follow a self-limiting clinical course, in certain populations, EBV can lead to a range of lymphoproliferative disorders (LPDs), particularly subtypes originating from T cells and natural killer (NK) cells, which are often characterized by highly aggressive disease progression. This review aims to systematically discuss the molecular basis of EBV infection, covering its viral biological properties, regulation of the latent and lytic cycles, key viral protein functions (e.g., LMP1, LMP2A, EBNA1), miRNA regulatory mechanisms, and the activation of various host signaling pathways (such as NF-κB, PI3K-AKT, JAK-STAT) that contribute to the maintenance of latent infection, cell transformation, and immune evasion. Additionally, the review focuses on the pathogenic contributions of these mechanisms in EBV-related T/NK cell lymphoproliferative diseases. Research highlights include the in-depth analysis of virus-host genome interaction mechanisms, the identification of novel molecular biomarkers, and the development of targeted therapeutic strategies (e.g., PD-1/PD-L1 immune checkpoint inhibitors, EBV-specific T cell therapy). Through this comprehensive review, it is hoped that personalized medicine and artificial intelligence-assisted multimodal decision-making will be applied to the precise prevention and treatment of EBV-related diseases.

## 1 Introduction

Epstein-Barr virus (EBV), also known as human gammaherpesvirus 4, is a lymphotropic double-stranded DNA virus with a genome of approximately 170 kb. In the current taxonomy framework of the International Committee on Taxonomy of Viruses, EBV is assigned to the order Herpesvirales, family Orthoherpesviridae, subfamily Gammaherpesvirinae, genus Lymphocryptovirus, and species Lymphocryptovirus humangamma4, with Epstein-Barr virus remaining the commonly used virus name (1). Recent clinicopathological and molecular studies have emphasized that EBV-positive T- and natural killer (NK)-cell lymphoproliferative disorders represent a heterogeneous disease spectrum, ranging from chronic inflammatory or proliferative disorders to highly aggressive lymphomas and leukemias (1). Epidemiological studies show that more than 90% of adults worldwide have been infected with EBV, and the virus can persist lifelong in the host, with B lymphocytes and epithelial cells being the best-characterized cellular reservoirs (2). In addition to these classical target cells, EBV can also be detected in T lymphocytes and NK cells, giving rise to a group of EBV-associated T/NK-cell lymphoproliferative disorders, including chronic active EBV infection (CAEBV), nasal-type NK/T-cell lymphoma (NKTCL), aggressive NK-cell leukemia (ANKL), and EBV-associated hemophagocytic lymphohistiocytosis (EBV-HLH) (3).

A critical issue in interpreting this disease spectrum is that EBV-related T/NK-cell disorders should not be viewed as a simple linear sequence from viral infection to malignancy. CAEBV is better understood as a chronic EBV-driven inflammatory and clonal proliferative disease of T/NK cells, in which only a subset of cases acquire additional host or viral genomic alterations and progress toward overt lymphoma or leukemia. EBV-HLH, in contrast, is primarily characterized by uncontrolled immune activation and cytokine storm triggered by EBV-infected lymphocytes, although it may coexist with or reveal an underlying clonal T/NK-cell disorder in selected patients. Therefore, this review focuses on EBV-positive T/NK-cell lymphoproliferative disorders and discusses B-cell or epithelial EBV-associated diseases only when they provide experimentally established mechanisms that help interpret T/NK-cell pathogenesis.

EBV is recognized as the first virus directly linked to human tumorigenesis. Its oncogenic and immune-modulating activities are mediated by a series of viral products, including latent membrane proteins such as LMP1 and LMP2A, Epstein-Barr nuclear antigens such as EBNA1, EBV-encoded small RNAs, viral miRNAs, and epigenetic regulatory mechanisms (4, 5). However, much of the mechanistic framework of EBV latency, receptor signaling mimicry, and viral protein function has been established in B-cell models. These mechanisms cannot be directly transposed to T/NK-cell diseases without qualification. For example, the role of LMP2A in mimicking B-cell receptor signaling is well supported in B cells, whereas its functional significance in T/NK-cell infection remains less clearly defined. Similarly, latency patterns traditionally classified as latency 0, I, II, and III are useful conceptual categories, but they should not be interpreted as rigid disease-specific programs in T/NK-cell lesions. NKTCL is often described as having a latency II-like pattern, yet genomic and transcriptomic analyses have shown attenuated and heterogeneous viral gene expression, with variable activation of latent and lytic programs across cases (6). This heterogeneity suggests that EBV adapts its transcriptional strategy according to host-cell lineage, immune pressure, and tissue microenvironment.

Advances in molecular detection have also reshaped the interpretation of EBV positivity and negativity. Conventional EBER *in situ* hybridization remains the routine standard for tissue-based EBV detection, and quantitative PCR is widely used for viral-load monitoring (7). Nevertheless, high-sensitivity approaches, including qPCR, droplet digital PCR, RNAscope-based EBNA1 mRNA detection, and refined *in situ* hybridization methods, have demonstrated low-level EBV nucleic acids in rare tumor cells of some lymphomas previously classified as EBV-negative by routine methods (8). Although these findings were mainly generated from B-cell lymphoma cohorts, they highlight a broader methodological limitation: EBV involvement may be underestimated when classification relies solely on conventional assays. This issue is particularly relevant for T/NK-cell lymphoproliferative disorders, where disease rarity, limited tissue availability, low or heterogeneous viral transcription, and variable tumor-cell content may reduce detection sensitivity. Incorporating high-sensitivity EBV detection into future studies may therefore refine disease classification, clarify the boundary between inflammatory EBV-driven proliferation and overt malignancy, and identify patient subsets in which viral persistence has been underrecognized.

Therefore, a comprehensive and cautious understanding of EBV infection in T/NK cells requires integration of viral taxonomy, detection methodology, latency heterogeneity, host-cell lineage specificity, and immune microenvironmental context. This review systematically discusses the molecular basis of EBV infection, signaling pathway regulation, immune evasion mechanisms, and their pathogenic roles in T/NK cell-derived lymphoproliferative disorders, with the aim of clarifying disease biology and identifying potential biomarkers and therapeutic targets.

## 2 Basic characteristics of Epstein-Barr virus

**Figure 1:**
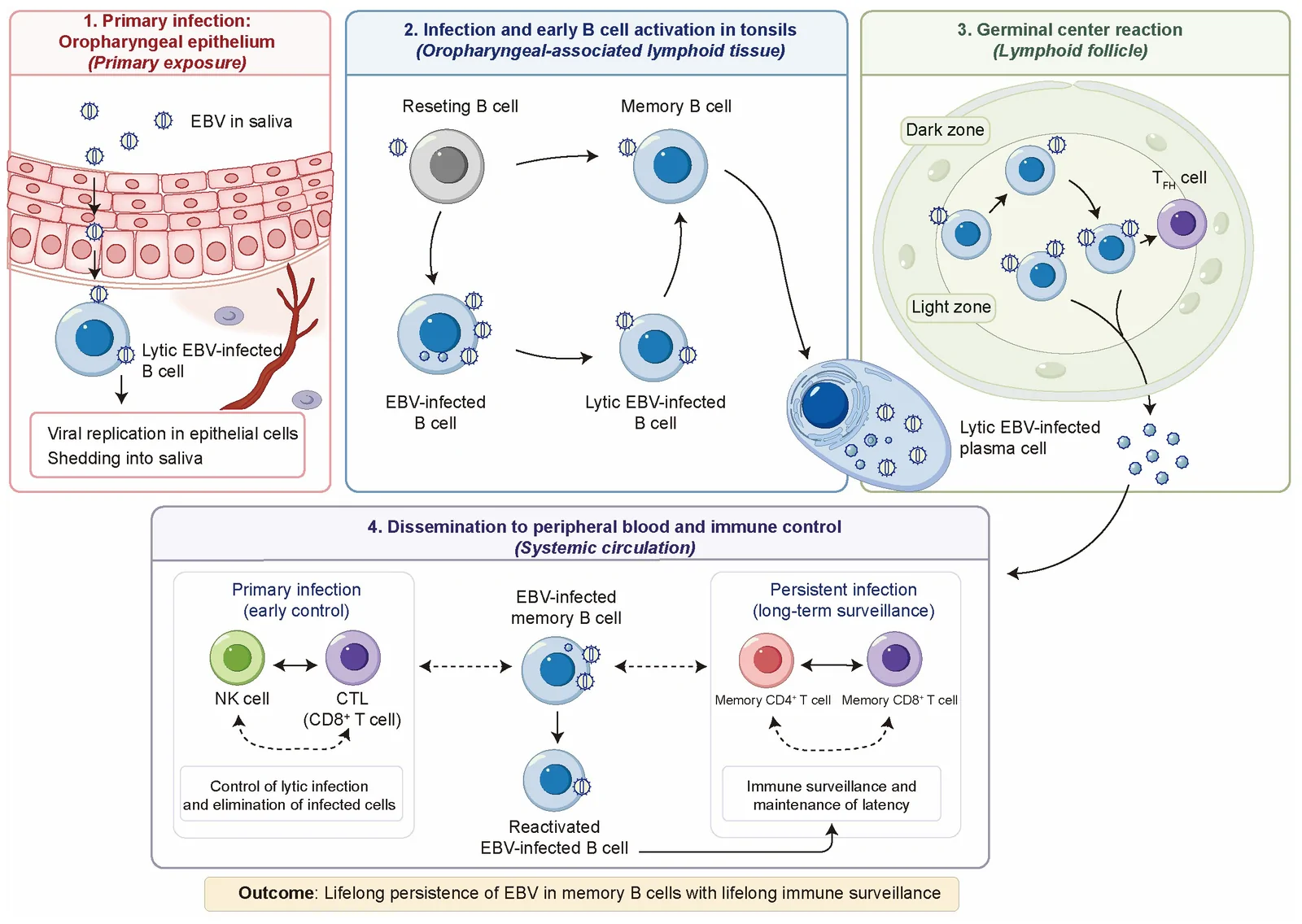
EBV primary infection, B-cell colonization, germinal-center passage, and immune-controlled lifelong persistence. EBV is mainly transmitted through saliva and initially encounters the oropharyngeal epithelium and oropharyngeal-associated lymphoid tissue. Viral replication in epithelial cells contributes to viral shedding into saliva, whereas infection of resting B cells in the tonsils promotes early B-cell activation. Infected B cells may enter germinal-center reactions, interact with follicular helper T cells, and differentiate either into memory B cells, which constitute the major long-term reservoir of latent EBV infection, or into plasma cells, in which lytic reactivation can produce infectious virions. Dissemination of EBV-infected memory B cells into the peripheral blood is controlled by innate and adaptive immune responses. NK cells and cytotoxic CD8+ T cells contribute to early control of lytic infection, whereas memory CD4+ and CD8+ T cells support long-term immune surveillance and maintenance of latency. The overall outcome is lifelong persistence of EBV in memory B cells with intermittent reactivation under immune control. This figure illustrates the canonical epithelial/B-cell model of EBV persistence, which provides a reference framework for interpreting EBV-associated T/NK-cell lymphoproliferative disorders.

Epstein-Barr virus (EBV) has a genome consisting of linear double-stranded DNA, approximately 170 kb in length. EBV is highly prevalent and infectious, making it one of the most widely distributed viruses in humans. Recent advances in molecular profiling and clinicopathological classification have further clarified that EBV can drive a broad spectrum of T/NK-cell lymphoproliferative disorders, including chronic active EBV infection, extranodal NK/T-cell lymphoma, aggressive NK-cell leukemia, EBV-associated hemophagocytic lymphohistiocytosis, and cutaneous EBV-positive T/NK-cell disorders (9). The canonical EBV life cycle has been defined mainly in B lymphocytes, and this model provides an important framework for understanding EBV persistence, although it cannot fully explain the biology of EBV infection in T/NK-cell diseases. Over 90% of adults worldwide are seropositive for EBV, with the virus primarily transmitted through saliva, and most individuals are infected during childhood. Although primary infection is typically asymptomatic, some individuals may develop infectious mononucleosis (IM), which usually follows a self-limiting clinical course. After infection, EBV can establish two types of infection states in the host: latent infection and lytic infection. In the classical B-cell model summarized in **Figure 1**, EBV is transmitted through saliva and first encounters the oropharyngeal epithelium and tonsillar lymphoid tissue. Viral replication in epithelial cells contributes to shedding into saliva, whereas infection of resting B cells in the tonsils promotes early B-cell activation and entry into germinal-center reactions. Infected B cells may differentiate into memory B cells, which serve as a long-term reservoir, or into plasma cells in which lytic reactivation releases infectious virions. During primary infection, NK cells and cytotoxic CD8+ T cells limit lytic infection and eliminate infected cells; during persistent infection, memory CD4+ and CD8+ T cells contribute to long-term immune surveillance. Thus, EBV persistence reflects a dynamic cycle of epithelial replication, B-cell latency, intermittent reactivation, and immune control (10, 11) (**Figure 1**).

**Figure 2:**
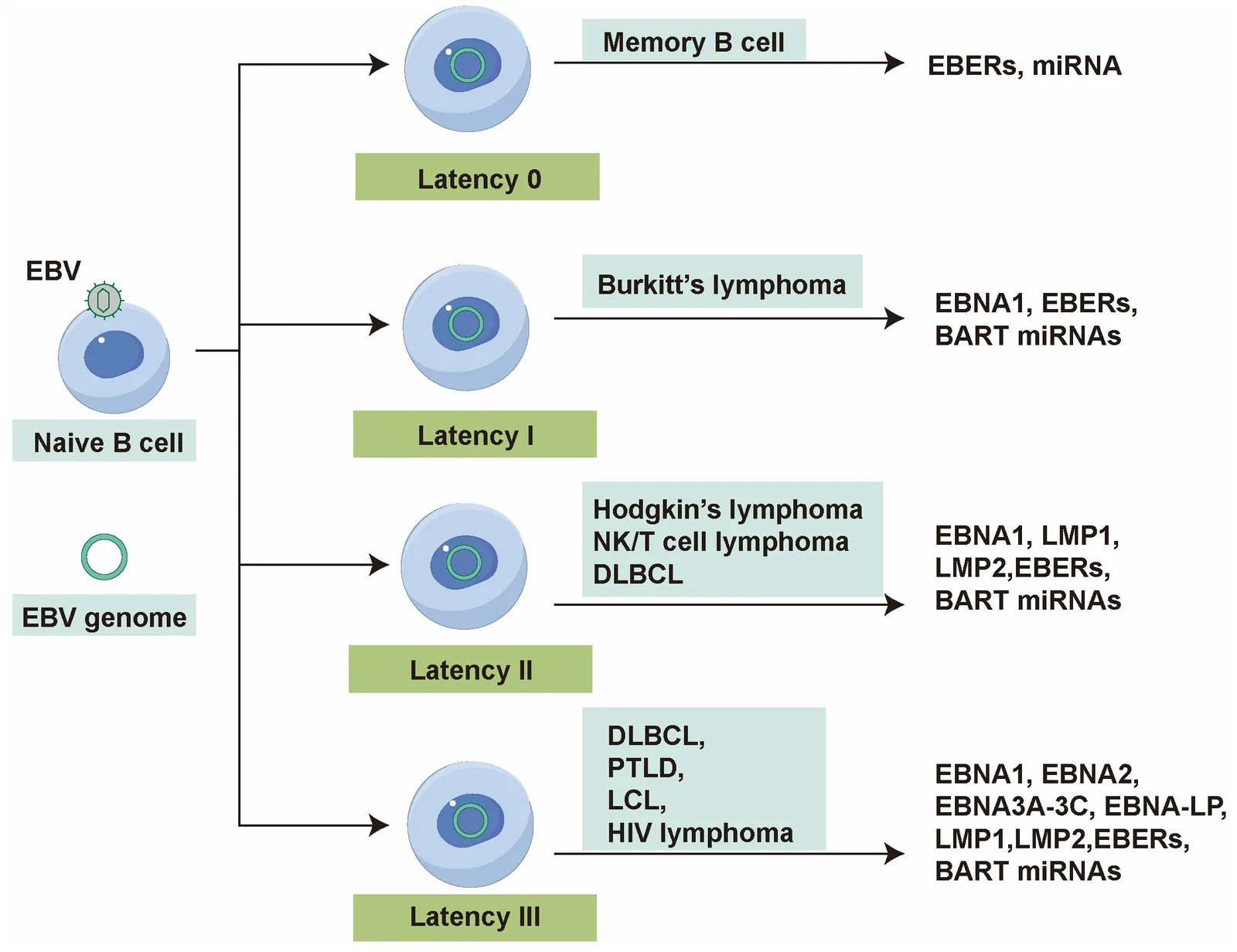
EBV latency programs and viral product expression across representative EBV-associated states. After infection of naive B cells, EBV persists as an episomal viral genome and establishes distinct latency programs characterized by different patterns of viral gene and non-coding RNA expression. Latency 0, typically associated with resting memory B cells, shows highly restricted viral transcription, mainly EBERs and viral miRNAs. Latency I, represented by Burkitt lymphoma, is characterized by EBNA1, EBERs, and BART miRNAs. Latency II, observed in classical Hodgkin lymphoma, NK/T-cell lymphoma, and subsets of diffuse large B-cell lymphoma, includes EBNA1, LMP1, LMP2, EBERs, and BART miRNAs. Latency III, seen in highly immunogenic or immunodeficiency-associated settings such as post-transplant lymphoproliferative disorder, lymphoblastoid cell lines, HIV-associated lymphoma, and some diffuse large B-cell lymphomas, expresses EBNA1, EBNA2, EBNA3A–3C, EBNA-LP, LMP1, LMP2, EBERs, and BART miRNAs. These latency categories are schematic operational patterns rather than rigid disease-specific states; in EBV-positive T/NK-cell lesions, viral transcription may be attenuated, heterogeneous, and influenced by host-cell lineage and immune microenvironmental pressure.

Viral entry into B cells is dependent on the primary envelope glycoprotein gp350 binding to the cell surface complement receptor 2 (CR2/CD21), with the auxiliary involvement of gp42 and HLA class II molecules to facilitate cell fusion. This receptor-dependent entry pathway is well established in B cells, but it should not be assumed to operate identically in T or NK cells. EBV infection of T/NK cells has been consistently documented in several EBV-positive T/NK-cell lymphoproliferative disorders, whereas the precise mechanisms of viral entry, transfer, and persistence in these lineages remain incompletely characterized (12–16). Proposed explanations include transient or aberrant expression of viral entry-related molecules, cell-to-cell transfer from infected B cells or epithelial cells, and lineage-specific survival advantages of infected T/NK-cell clones. Therefore, B-cell infection provides an essential reference model, but the interpretation of EBV biology in T/NK-cell disorders requires disease- and lineage-specific caution. Long-term latent infection predominantly resides in memory B cells, helping the virus evade immune surveillance (17). As summarized in **Figure 2**, EBV latency is conventionally classified into latency 0, I, II, and III, each characterized by progressively broader viral product expression. Latency 0 in memory B cells is associated with minimal viral transcription, mainly EBERs and viral miRNAs; latency I, exemplified by Burkitt lymphoma, typically expresses EBNA1, EBERs, and BART miRNAs; Latency II, observed in classical Hodgkin lymphoma, NK/T-cell lymphoma, and subsets of diffuse large B-cell lymphoma, includes EBNA1, LMP1, LMP2, EBERs, and BART miRNAs; and latency III, seen in highly immunogenic states such as post-transplant lymphoproliferative disorder, lymphoblastoid cell lines, HIV-associated lymphoma, and some diffuse large B-cell lymphomas, expresses the full EBNA program together with latent membrane proteins, EBERs, and BART miRNAs (**Figure 2**) (17). However, these latency categories should be regarded as operational patterns rather than fixed disease-specific states, particularly in EBV-positive T/NK-cell lesions. For example, extranodal NK/T-cell lymphoma is often described as latency II-like, but genomic and transcriptomic studies have shown heterogeneous viral transcription, attenuated latent gene expression, abundant BART miRNAs, and variable expression of selected lytic genes across cases (6). Thus, assigning a single latency program to a given T/NK-cell disease may oversimplify the dynamic interaction between EBV, host-cell lineage, and the immune microenvironment. In contrast, during lytic infection, the virus completes full genomic expression and releases mature viral particles. The lytic cycle can be divided into five stages: immediate-early (IE), early, late gene expression, virus assembly, and release (18, 19). The transcription factors BZLF1 and BRLF1 are key inducing proteins that cooperatively activate the expression of early lytic genes, initiating viral replication and subsequent viral particle assembly. Since its discovery, EBV has been linked to multiple human malignancies, particularly under conditions of immune dysregulation. Its pathogenic role is firmly established in B-cell lymphoproliferative diseases such as Burkitt lymphoma and classical Hodgkin lymphoma; however, these diseases serve mainly as mechanistic comparators in the present review. In T/NK-cell lymphoproliferative disorders, EBV is consistently associated with disease initiation or progression, but the underlying biology is less completely defined than in B-cell models (12–16). Available evidence indicates that EBV infection in T/NK cells involves lineage-specific viral transcription, host immune activation, clonal expansion, and acquisition of additional molecular abnormalities. The following sections therefore focus on EBV-associated T/NK-cell diseases, while distinguishing mechanisms directly supported by T/NK-cell data from those inferred from better-characterized B-cell or epithelial models.

## 3 Molecular mechanisms of EBV infection in NK/T cells

EBV infection in NK/T cells involves multiple viral and host mechanisms, including viral protein-mediated signaling regulation, post-transcriptional modulation by viral miRNAs, and aberrant activation of several host signaling pathways, such as NF-κB, PI3K-AKT, and JAK-STAT. However, the strength of evidence differs among these mechanisms. Some findings are directly supported by studies of EBV-positive T/NK-cell diseases, whereas others are inferred from better-characterized B-cell or epithelial models. Collectively, these mechanisms may contribute to the continuous proliferation, anti-apoptotic activity, immune evasion, and clonal expansion of EBV-infected cells, providing a molecular foundation for the development of EBV-related T/NK cell diseases (**Table 1**).

**Table 1:** Overview of molecular mechanisms of EBV infection in NK/T cells.

Category	Name	Functional description
Viral Proteins	LMP1	Mimics CD40 signaling, activates NF-κB, PI3K, and MAPK pathways, promotes cell proliferation and survival, induces immune evasion
Viral Proteins	LMP2A/LMP2B	Promotes B cell survival, prevents viral lytic replication, mimics BCR signaling, maintains latent viral infection
Viral Proteins	EBNA1	Maintains viral genome stability, regulates viral and host gene expression, inhibits apoptosis, involved in immune evasion
Viral Proteins	EBNA2	Activates transcription of viral and host genes, promotes B cell immortalization, suppresses host immune response
Viral Proteins	EBNA3 Family	Regulates transcription of viral and host genes, maintains latent viral infection, influences cell proliferation and apoptosis
miRNA Regulation	BART miRNA	Inhibits apoptosis, modulates immune response, maintains latent viral infection, promotes tumorigenesis
miRNA Regulation	BHRF1 miRNA	Regulates apoptosis, suppresses host immune response, supports viral particle production
Signaling Pathway Activation	NF-κB Pathway	Promotes cell proliferation, production of inflammatory cytokines, maintains latent viral infection
Signaling Pathway Activation	JAK-STAT Pathway	Regulates immune response, promotes cell survival and proliferation, shapes the tumor microenvironment
Signaling Pathway Activation	PI3K-AKT Pathway	Promotes cell metabolism, anti-apoptosis, supports latent viral infection, associated with tumor resistance
Immune Evasion Mechanism	PD-L1 Expression	Upregulates PD-L1 through various mechanisms, inhibits T cell activity, promotes immune evasion
Immune Evasion Mechanism	MHC-I Downregulation	Inhibits MHC-I molecule expression through various mechanisms, evades immune surveillance

### 3.1 Role of latent viral proteins

EBV latent proteins play a central role in maintaining infection, driving cell transformation, and regulating host signaling networks. Among them, latent membrane proteins (LMP1, LMP2A) and Epstein-Barr nuclear antigens (EBNA) family members (especially EBNA1, EBNA2, and EBNA3 subtypes) are the most representative pathogenic molecules. Although these proteins were initially characterized in B-cell models, available evidence suggests that some of their downstream effects may be retained, modified, or reprogrammed in EBV-positive T/NK-cell lesions. Among these latent proteins, LMP1 and EBNA1 have relatively stronger relevance to T/NK-cell diseases, whereas the roles of LMP2A, EBNA2, and EBNA3 family members are mainly supported by B-cell models or selected EBV-related tumors.

#### 3.1.1 LMP1: a core driver of signaling and oncogenesis

Latent membrane protein 1 (LMP1) is considered the most important oncogene of EBV. This protein is composed of 386 amino acids and contains six transmembrane domains and a functional C-terminal cytoplasmic domain, which includes three signaling active regions (CTAR1, CTAR2, and CTAR3). LMP1 mimics the signal transduction process of CD40, a member of the TNF receptor family, persistently recruiting TNF receptor-associated factors (TRAFs) to activate multiple pro-survival and pro-inflammatory pathways, including NF-κB, JNK, p38 MAPK, and PI3K-AKT (20–26). This continuous signaling activation not only confers anti-apoptotic capabilities to the infected cells but also promotes alterations in their immune regulatory functions. In EBV-positive T/NK-cell lesions in which LMP1 is expressed, LMP1 may contribute to immune-microenvironment remodeling through NF-κB-dependent inflammatory signaling and cytokine-related regulation. IL-9 expression and viral IL-10 expression have also been reported in NK/T-cell lymphoma contexts, supporting the concept that EBV-positive lesions can shape a cytokine-rich microenvironment (27, 28). Additionally, LMP1-driven activation of NF-κB and related STAT/AP-1 signaling can upregulate PD-L1 expression, thereby impairing T cell-mediated immune clearance (29, 30). This “signal camouflage” may contribute to the maintenance of EBV latency and immune escape, although its relative importance may vary across different T/NK-cell disease entities.

#### 3.1.2 LMP2A: a latency maintenance factor mimicking receptor signals

Latent membrane protein 2 (LMP2) exists in two forms, LMP2A and LMP2B, with LMP2A possessing a complete signaling domain. Its N-terminal cytoplasmic region contains an immunoreceptor tyrosine-based activation motif (ITAM), which recruits Syk and Src family tyrosine kinases, thereby mimicking the downstream signaling of the B cell receptor (BCR) (31–36). Although the function of LMP2A was initially characterized in B cells, direct evidence for an equivalent receptor-mimicking role in T/NK-cell infection remains limited. In EBV-positive T/NK-cell diseases, LMP2A expression should therefore be interpreted as part of a heterogeneous latent transcription program rather than as a uniformly active driver. When expressed, LMP2A may contribute to survival signaling through PI3K-AKT- and ERK-related pathways, but whether this reflects direct signaling in T/NK cells or extrapolation from B-cell models requires further validation. Thus, LMP2A is best regarded as a potential latency-associated modulator, rather than a fully established oncogenic mechanism in T/NK-cell disorders.

#### 3.1.3 EBNA family: a key regulatory layer in genome stability and immune evasion

The Epstein-Barr nuclear antigens (EBNAs) family plays a central role in the maintenance of viral latency and host gene reprogramming. Among them, EBNA1 is expressed in all EBV-associated tumors and is a core regulator of viral genome maintenance and replication. EBNA1 binds to the viral replication origin (oriP) region, mediating the replication of the viral DNA and its partitioning into the host chromosome (37–43). Additionally, its glycine-alanine repeat region (GAr) suppresses its own translation, reducing antigen processing and MHC I molecule presentation, thereby effectively evading immune surveillance (44–46). Furthermore, EBNA1 can downregulate NKG2D ligand expression, thereby reducing NK cell-mediated cytotoxicity, although this mechanism has been mainly demonstrated in B-cell infection models (47). These features make EBNA1 a key molecule in maintaining the “silent presence” of EBV during latency. EBNA2 is a major transcriptional activator during B-cell latency and regulates the expression of various viral and host genes, including LMP1 and LMP2, through interaction with the transcription factor RBP-Jκ (48–53). However, its expression and functional contribution in EBV-positive T/NK-cell lesions are less consistent than those in B-cell latency models. Therefore, EBNA2-related transcriptional regulation should be considered a mechanistic reference rather than a universally established pathway in T/NK-cell infection. Similarly, the EBNA3 family members, including EBNA3A, EBNA3B, and EBNA3C, have been shown mainly in B-cell systems to regulate host transcription and epigenetic programs. EBNA3C can silence host tumor suppressor genes by interacting with Polycomb-related regulatory complexes, while loss of EBNA3B has been associated with enhanced oncogenic potential in experimental models (54–57). These findings provide important insight into EBV-driven host-cell reprogramming, but their direct relevance to T/NK-cell lymphoproliferative disorders requires further disease-specific validation.

Overall, EBV latent proteins participate in viral genome maintenance, immune evasion, and host-cell signaling regulation, but their contribution differs among cell lineages and disease entities. In T/NK-cell lymphoproliferative disorders, LMP1 and EBNA1 appear to have more consistent biological relevance, whereas LMP2A-, EBNA2-, and EBNA3-related mechanisms should be interpreted more cautiously because much of the current evidence derives from B-cell models. Unlike the classical B-cell model, EBV infection in T/NK cells tends to be associated with chronic inflammatory proliferation, cytokine imbalance, and clonal evolution rather than typical B-cell immortalization. This difference suggests that EBV pathogenesis in T/NK cells is not a simple replication of B-cell latency biology, but instead depends on lineage-specific reconfiguration of host signaling pathways. This understanding provides a theoretical basis for explaining the unique pathological features of CAEBV and NK/T-cell lymphoma, among other diseases.

### 3.2 Regulatory mechanisms of miRNA

In addition to encoding protein products, EBV expresses various non-coding small RNAs, particularly microRNAs (miRNAs), during its latent phase. These miRNAs play a crucial post-transcriptional regulatory role in the molecular interactions between the virus and host cells. To date, 25 EBV-encoded miRNA precursors have been identified, which can be processed into 44 mature viral miRNAs. These miRNAs are primarily classified into two major groups based on their position in the viral genome: BART (BamHI A rightward transcripts) miRNAs and BHRF1 (BamHI fragment H rightward open reading frame 1) miRNAs (58, 59). Generally, BART miRNAs are broadly expressed in many EBV-positive cellular contexts, especially in epithelial cells and some T/NK-cell lesions, where they are relatively stable. In contrast, BHRF1 miRNAs are primarily expressed during latency phase III and are commonly found in B cells, correlating with high transformation potential. There are significant differences in the expression profiles and functional mechanisms of these two miRNA classes in different cell types, suggesting that EBV may modulate its infection strategy and pathogenic pathways through specific miRNA modules (60, 61).

#### 3.2.1 BART miRNAs

BART miRNAs are widely involved in reshaping the biological behavior of host cells, particularly in regulating apoptosis and immune responses. Several BART miRNAs target host pro-apoptotic factors, blocking mitochondrial pathways or the expression of lytic-related genes. For example, miR-BART1-3p and miR-BART16 inhibit the expression of Caspase-3, blocking execution-phase apoptosis (62). miR-BART20-5p targets the viral genes BRLF1, BZLF1, and the cytokine BAD, preventing the initiation of EBV lytic cycle (63, 64). Furthermore, miR-BART4-5p and miR-BART15 significantly reduce mitochondrial-mediated apoptosis by inhibiting Bcl-2 family proteins such as BIM and BID (65–67). miR-BART16 targets the mitochondrial membrane transporter TOMM22, interfering with Bax localization, thereby stabilizing host cell survival (68). These miRNA functions synergize to provide a molecular basis for the long-term maintenance of EBV latency in T/NK cells. In immune regulation, some BART miRNAs also inhibit CD8+ and CD4+ T cell-mediated cytotoxic responses, enhancing the immune evasion of infected cells (62, 66, 69, 70). For instance, miR-BART17-5p has been reported to downregulate the expression of MHC class I molecules, thereby reducing the efficiency of viral antigen presentation. These mechanisms may be particularly relevant in T/NK-cell diseases, because immune surveillance and cytotoxic function are central features of these lineages; however, systematic functional validation in T/NK-cell models remains limited.

#### 3.2.2 BHRF1 miRNAs

In contrast, BHRF1 miRNAs primarily support the early stages of viral infection and replication. These miRNAs (such as miR-BHRF1-1, -2, and -3) inhibit apoptosis by upregulating BHRF1 protein expression, thereby enhancing host cell survival (71, 72). Notably, their expression increases during early infection but decreases after the virus stabilizes in latency, suggesting that they play a temporally specific role in regulating the viral infection phase, or they may help “silently” maintain latency to evade immune system detection. Recent studies show that miR-BHRF1-2-5p can target the interleukin-1 receptor (IL1R1), suppressing NF-κB pathway activation and reducing the release of inflammatory cytokines such as IL-1A, IL-1B, and IL-6, thereby mitigating the amplification of inflammatory signals (73). Additionally, this miRNA can target multiple host factors involved in immune signal transduction, such as BIRC3, PLCG1, and SOS1, further interfering with the cytokine network. Another representative molecule, miR-BHRF1-1, downregulates the SUMO ubiquitin ligase RNF4, inhibiting the degradation of proteins involved in viral clearance and enhancing viral particle replication and release capacity (74). EBV-encoded miRNAs are key molecules that maintain “silent but biologically active” states during latent infection, establishing a regulatory system independent of protein expression. In T/NK cells, where classical BCR-dependent latency mechanisms are absent, viral miRNAs may represent an especially important layer of latency regulation and immune evasion, although their disease-specific functions require further experimental clarification.

From a clinical perspective, the cell-type-specific expression profiles of BART and BHRF1 miRNAs may provide complementary biomarkers for disease diagnosis, classification, and therapeutic monitoring, although validation in T/NK-cell disease-specific cohorts remains limited (58–61). Moreover, EBV-encoded miRNAs themselves may represent potential therapeutic intervention targets, especially in T/NK-cell diseases where traditional protein-targeted therapies are difficult to apply (62, 66, 69, 70). Their therapeutic value warrants further exploration.

### 3.3 Activation of key signaling pathways

**Figure 3:**
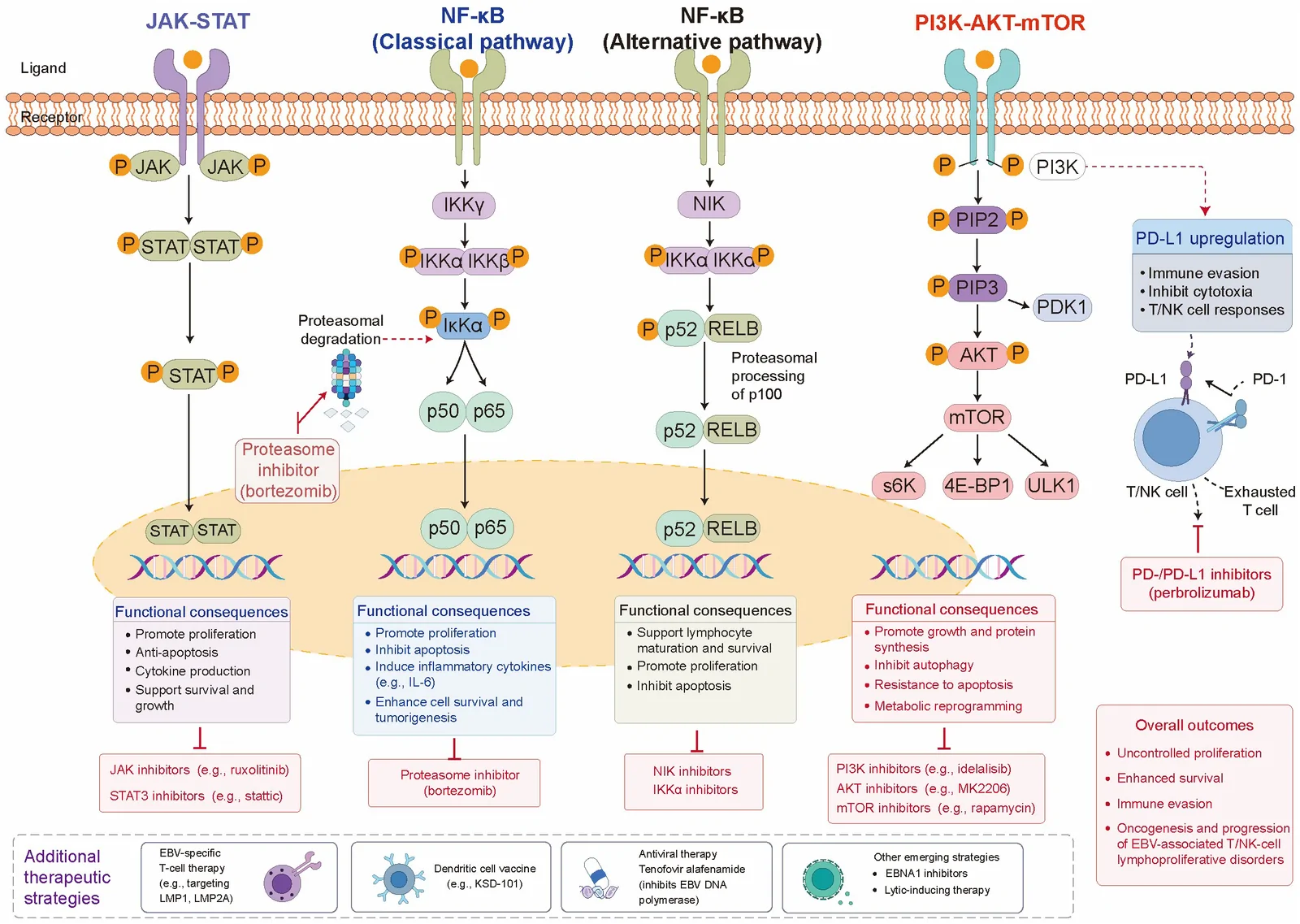
Key signaling pathways, immune-evasion mechanisms, and therapeutically targetable nodes in EBV-associated T/NK-cell lymphoproliferative disorders. The diagram summarizes major host signaling axes implicated in EBV-associated T/NK-cell disease biology. The JAK-STAT pathway is activated by cytokine-receptor signaling and promotes STAT phosphorylation, nuclear translocation, proliferation, cytokine production, and cell survival; potential inhibitors include JAK inhibitors such as ruxolitinib and STAT3 inhibitors. The canonical NF-κB pathway involves IKK-mediated IκBα phosphorylation and proteasomal degradation, leading to p50/p65 nuclear translocation, inflammatory cytokine induction, enhanced survival, and tumorigenesis; proteasome inhibitors such as bortezomib may interfere with this axis. The alternative NF-κB pathway involves NIK, IKKα, p100 processing, and p52/RelB activation, supporting lymphocyte maturation, survival, and proliferation; NIK and IKKα are potential therapeutic targets. The PI3K-AKT-mTOR pathway promotes growth, protein synthesis, resistance to apoptosis, inhibition of autophagy, and metabolic reprogramming, and may be targeted by PI3K, AKT, or mTOR inhibitors such as idelalisib, MK2206, and rapamycin. These signaling pathways can also converge on PD-L1 upregulation, enabling immune evasion through PD-1 engagement on exhausted T cells and inhibition of cytotoxic T/NK-cell responses; PD-1/PD-L1 inhibitors such as pembrolizumab represent clinically relevant immunotherapeutic strategies. Additional emerging approaches include EBV-specific T-cell therapy targeting latent antigens such as LMP1 and LMP2A, dendritic cell vaccines such as KSD-101, antiviral therapy such as tenofovir alafenamide, EBNA1 inhibitors, and lytic-induction strategies. Together, these pathways support uncontrolled proliferation, enhanced survival, immune evasion, and progression of EBV-associated T/NK-cell lymphoproliferative disorders, although the disease-specific relevance of each target requires further validation.

EBV infection activates multiple key signaling pathways in host cells, altering cell fate programs, helping the virus establish latency, and evading immune clearance. In T/NK cells, aberrant activation of these pathways not only supports the maintenance of viral infection but may also trigger malignant transformation and reshape the immune microenvironment. Current research focuses on interconnected signaling and immune-regulatory axes, including JAK-STAT, canonical and non-canonical NF-κB, PI3K-AKT-mTOR, and PD-L1-mediated immune checkpoint regulation, as well as therapeutically targetable nodes within these pathways (**Figure 3**).

#### 3.3.1 NF-κB pathway

NF-κB is a critical transcription factor family involved in regulating cell inflammation, immune response, and survival. This pathway can be activated through two major modes: the canonical and non-canonical pathways (75). The canonical pathway is activated by stimuli such as TNF-α, IL-1β, pathogen-associated molecular patterns (PAMPs), or bacterial lipopolysaccharides (LPS). This leads to the activation of the IKK complex, resulting in the phosphorylation and degradation of IκB, releasing NF-κB to enter the nucleus and initiate the transcription of inflammation and survival genes (76–81). The non-canonical pathway is activated by stimuli such as CD40, BAFF, or lymphotoxin β (LTβ), which activate NF-κB-inducing kinase (NIK) and IKKα, mediating the cleavage of p100 protein to p52, which then forms dimers with RelB to exert its function (82–85). In the context of EBV infection, LMP1 is one of the best-characterized viral activators of the NF-κB pathway. LMP1 mimics CD40 signaling and continuously activates NF-κB, not only conferring anti-apoptotic properties to the infected cell but also upregulating pro-inflammatory factors (e.g., IL-6, TNF-α) and immune regulatory molecules (e.g., PD-L1) (86, 87). In EBV-related T/NK cell diseases, the sustained activation of this pathway may be a key link between viral latency, the maintenance of the inflammatory microenvironment, and tumorigenesis.

#### 3.3.2 JAK-STAT pathway

The JAK-STAT pathway is an evolutionarily conserved cytokine signaling pathway widely involved in immune response, cell proliferation, and metabolic regulation (88, 89). This pathway is mediated by the JAK family of tyrosine kinases (JAK1, JAK2, JAK3, TYK2), which phosphorylate receptors and subsequently activate STAT family transcription factors (e.g., STAT1, STAT3) to enter the nucleus and regulate gene expression (90, 91). In normal immune environments, the JAK-STAT pathway promotes viral clearance through the interferon-STAT1 axis and regulates inflammation and immune suppression via the IL-6-STAT3 axis (92). NK cell function also heavily relies on IL-2, IL-15, and IFN-mediated STAT signaling activation (93–96). EBV-positive T/NK-cell lesions often show activation of STAT-related signaling, which may be driven by viral products such as LMP1, cytokine stimulation, and host genetic alterations, leading to abnormal inflammatory gene expression and immune-evasion programs (30, 97–101). In CAEBV and NK/T-cell lymphoma, persistent JAK/STAT activation has been associated with cell survival, inflammatory signaling, PD-L1 induction, and disease progression, supporting this axis as a potentially actionable therapeutic pathway (102).

#### 3.3.3 PI3K-AKT pathway

The PI3K-AKT pathway also plays a crucial role in cell metabolism, proliferation, and anti-apoptosis, making it one of the key signaling axes hijacked by EBV in infected cells (103, 104). In terms of metabolic regulation, the PI3K-AKT pathway activates mTORC1, promoting the synthesis of proteins, lipids, and nucleic acids to meet the biosynthetic and energy needs of rapidly proliferating cells (105, 106). In cell survival, AKT inactivates pro-apoptotic proteins such as BAD and BAX by phosphorylation and upregulates the expression of anti-apoptotic proteins like Bcl-xL, Bcl-2, and Mcl-1, significantly enhancing the cell’s tolerance to various stress signals (107). Numerous studies have shown that in EBV-related malignancies, the sustained activation of the PI3K-AKT pathway is primarily mediated by latent membrane proteins LMP1 and LMP2A (108). By activating the PI3K-AKT pathway, EBV acts on multiple downstream targets, increasing genomic instability, enhancing cell proliferation, inhibiting apoptosis, and remodeling cytoskeletal dynamics, leading to various oncogenic phenotypes (109). Evidence from other EBV-associated tumors, including nasopharyngeal carcinoma and classical Hodgkin lymphoma, supports the broader role of EBV latent proteins in activating PI3K-AKT-related survival signaling (108). However, in EBV-positive T/NK-cell lymphoproliferative disorders, this pathway should be discussed mainly as a survival and inflammatory signaling node rather than as a mechanism uniformly proven across all EBV-associated diseases. These findings collectively indicate that the PI3K-AKT pathway may contribute to EBV-induced abnormal proliferation and survival of T/NK cells and may provide a potential therapeutic target for subsequent interventions.

In summary, EBV infection is associated with the activation of multiple signaling networks in host cells, forming a proliferation, anti-apoptosis, and immune regulation axis centered on NF-κB, JAK-STAT, and PI3K-AKT pathways. In T/NK-cell diseases, these pathways are not driven exclusively by viral proteins, but may also be shaped by cytokine stimulation, immune microenvironmental pressure, and host genetic alterations. The synergistic activation of these pathways may support viral persistence, inflammatory amplification, tumor microenvironment remodeling, and immune evasion. Future studies should further distinguish virus-driven signaling from host-cell-intrinsic oncogenic events, which will be essential for developing precise pathway-targeted therapies.

### 3.4 Immune evasion mechanisms

Throughout its long coexistence with the host, EBV has evolved a highly complex immune evasion strategy to avoid recognition and clearance by T cells and NK cells. Immune evasion not only aids in the maintenance of viral latency but also provides support for the development of virus-associated tumors by shaping the microenvironment. EBV achieves immune evasion through multiple mechanisms, among which PD-L1 upregulation and MHC-I downregulation are two well-characterized strategies. The signaling connections among JAK-STAT, NF-κB, PI3K-AKT-mTOR, PD-L1 upregulation, and emerging targeted or immune-based interventions are summarized in **Figure 3**.

#### 3.4.1 Upregulation of PD-L1 expression

Programmed death-1 (PD-1) and its ligand PD-L1 (programmed death-ligand 1) are crucial immune checkpoint molecules that regulate T cell function. High expression of PD-L1 can bind to PD-1, inhibiting T cell activation and cytotoxic responses, thus creating an “immune cold microenvironment” (110–112). In EBV infection, various viral-encoded products directly or indirectly induce PD-L1 expression:

#### 3.4.2 Viral protein-mediated regulation

LMP1 significantly upregulates PD-L1 expression by activating the JAK/STAT, NF-κB, and AP-1 pathways, especially in NK/T-cell lymphoma tissues, where its expression is closely related to LMP1 activity (29, 30). EBNA1 enhances PD-L1 promoter activity through the JAK2/STAT1/IRF-1 axis (113). In B-cell models, EBNA2 suppresses the transcription factor EBF1, indirectly downregulating miR-34a and releasing the negative regulation on PD-L1 expression (114). Whether this regulatory axis operates similarly in EBV-positive T/NK-cell diseases remains to be clarified.

#### 3.4.3 Viral non-coding RNA

EBV-miR-BHRF1-2-5p binds to the 3’-UTR of PD-L1 and downregulates its expression, indicating that the virus may finely regulate PD-L1 at different stages (115). In epithelial tumor models, circRNA CircBART2.2 activates the RIG-I signaling pathway and can upregulate PD-L1, suggesting that EBV non-coding RNAs may regulate immune checkpoints in a context-dependent manner (116).

#### 3.4.4 Tumor microenvironment remodeling

Not only tumor cells but also antigen-presenting cells (e.g., dendritic cells, macrophages) express PD-L1. Under the influence of cytokines such as IFN-γ, IL-2, and IL-10, a synergistic immune suppressive state is formed (117–119). Studies show that EBV-encoded miR-BART11 and miR-BART17-3p enhance IFN-γ-induced PD-L1 expression (113). These mechanisms collectively build a “PD-L1 high-expression barrier,” allowing EBV-infected cells to evade T cell-mediated immune surveillance, especially in CAEBV and NK/T-cell lymphoma.

#### 3.4.5 Downregulation of MHC-I expression

MHC-I molecules (including HLA-A, HLA-B, HLA-C) are essential for CD8+ T cell and NK cell recognition of viral antigens. EBV uses multiple mechanisms to downregulate MHC-I expression or function, thereby inhibiting antigen presentation and evading cytotoxic immune recognition (120–122):

#### 3.4.6 Viral structural proteins mask antigen expression

EBV late protein gp150 forms a high-density glycosylation barrier on the cell surface, physically blocking the expression and function of MHC-I, MHC-II, and CD1d molecules (123).

#### 3.4.7 Interference with antigen processing and transport

The transporter associated with antigen processing (TAP) carries degraded peptides to the endoplasmic reticulum to bind to MHC-I. EBV’s BNLF2a protein interacts with TAP, blocking its transport function and significantly reducing antigen presentation efficiency (124, 125).

#### 3.4.8 Targeting the MHC-I degradation pathway

BDLF3 promotes the ubiquitination and degradation of MHC-I molecules, further reducing their surface expression and weakening T cell recognition (126).

#### 3.4.9 Coordinated inhibition by latent proteins and miRNAs

EBNA1 blocks the processing of its own antigens and MHC presentation through its GAr repeat region (127), while miRNAs targeting TAP2 expression exacerbate MHC-I downregulation. LMP1 and LMP2 indirectly affect immune cell activity and MHC-related pathways, amplifying immune evasion effects (47, 128).

#### 3.4.10 Dendritic cell function inhibition

EBV infection of plasmacytoid dendritic cells (pDCs) prevents their maturation, reducing type I interferon production and further impairing innate immune responses (129).

These mechanisms may contribute to viral persistence and immune escape, but their relative importance in T/NK-cell diseases likely varies according to disease subtype, viral gene expression pattern, tumor-cell content, and host immune pressure. EBV’s immune evasion mechanisms exhibit “multi-layered, multi-target, dynamic regulation,” not only regulating PD-L1 expression to create an “immune suppressive barrier” but also interfering with antigen processing and presentation to create an “antigen-deficient phenotype,” enabling dual evasion from CD8+ T cells and NK cells. In EBV-related diseases derived from T/NK cells, where innate immune activity is stronger, the virus relies on more efficient immune evasion mechanisms to maintain latency. From a clinical perspective, the abnormal activation of the PD-1/PD-L1 axis has become an important therapeutic target for NK/T-cell lymphoma. PD-1 inhibitors, such as Pembrolizumab and Nivolumab, have demonstrated significant efficacy in these patients. Future combination strategies, such as MHC-I restoration (e.g., TAP repair or EBNA1 antigen presentation activation), could further disrupt EBV-induced immune silencing and improve patient prognosis.

## 4 EBV-associated NK/T-cell lymphoproliferative disorders

Epstein-Barr Virus (EBV) can infect T lymphocytes and natural killer (NK) cells, leading to a variety of lymphoproliferative diseases (LPDs) originating from T/NK cells. These diseases are typically characterized by high invasiveness, resistance to treatment, and significant heterogeneity, including chronic active EBV infection (CAEBV), nasal-type NK/T-cell lymphoma (NKTCL), aggressive NK-cell leukemia (ANKL), and EBV-associated hemophagocytic lymphohistiocytosis (EBV-HLH). Below, the molecular features and pathogenic mechanisms of each major disease type will be discussed. To provide a clearer comparative framework across the EBV-associated T/NK-cell disease spectrum, **Table 2** integrates the major entities discussed in this review with their dominant signaling programs, recurrent genetic or viral alterations, current diagnostic approaches, and emerging therapeutic targets.

**Table 2:** Integrated summary of major EBV-associated NK/T-cell lymphoproliferative disorders, molecular features, diagnostic approaches, and therapeutic targets.

Disorder/entity	Key activated signaling pathways	Recurrent genetic or viral alterations	Currently used diagnostic biomarkers/approaches	Emerging or proposed therapeutic targets
Chronic active EBV disease/infection (CAEBV), T- or NK-cell type	Persistent NF-κB and JAK/STAT3 activation; cytokine-driven IL-6/IL-10/IFN-γ loops; PI3K–AKT–mTOR survival signaling.	Clonal expansion of EBV-infected T/NK cells; somatic DDX3X, STAT3 and KMT2D alterations in subsets; defective EBV genome, including BamHI A or lytic-gene deletions.	Persistent systemic inflammation and organ involvement; elevated EBV DNA load (updated guidelines propose ≥10,000 IU/mL in whole blood); EBER-ISH; lineage assignment by flow-FISH/sorted-cell EBV PCR; clonality and NGS when available.	Allogeneic HSCT with curative intent; JAK or mTOR inhibition as disease-control/bridging strategies; EBV-specific cytotoxic T cells; EBNA1/viral-miRNA and checkpoint-directed approaches in selected settings.
Extranodal NK/T-cell lymphoma (NKTCL/ENKTL)	JAK/STAT, PI3K–AKT–mTOR, MAPK and variably LMP1-related NF-κB signaling; PD-1/PD-L1 immune-evasion axis; EZH2/epigenetic programs.	DDX3X, TP53, STAT3/STAT5B/JAK3/JAK1 and PTPRK alterations; PRDM1/6q21 loss, BCOR, MGA and KMT2D changes; PD-L1/PD-L2 structural or copy-number alterations.	Extranodal destructive lesion; universal/near-universal EBER positivity; cytotoxic phenotype (CD2+, cytoplasmic CD3ϵ+, CD56+, granzyme B/perforin/TIA1+); plasma EBV DNA; PD-L1 IHC and NGS for risk stratification.	L-asparaginase-based regimens and radiotherapy in established practice; PD-1/PD-L1 blockade; JAK/STAT, PI3K–AKT–mTOR, EZH2/HDAC, CD30 and EBV-directed targets under investigation.
Aggressive NK-cell leukemia (ANKL)	JAK/STAT and RAS–MAPK signaling; NF-κB and PI3K–AKT survival pathways; BCL-2 dependence; PD-L1-mediated immune evasion in a subset.	STAT3/STAT5B/JAK2 alterations or JAK–STAT copy gains; RAS/MAPK-pathway mutations; DDX3X and TP53 alterations; epigenetic-modifier mutations.	Acute systemic leukemia-like presentation with hepatosplenomegaly and cytopenias; peripheral blood/bone marrow NK-cell proliferation; EBER positivity; CD2+, surface CD3−, cytoplasmic CD3ϵ+, CD56+ phenotype; EBV DNA and NGS.	L-asparaginase-containing chemotherapy and allogeneic HSCT when feasible; JAK inhibitors, BCL-2 inhibition, PD-1/PD-L1 blockade and EBV-specific cellular therapy as investigational or selected strategies.
EBV-associated hemophagocytic lymphohistiocytosis (EBV-HLH)	Th1/IFN-γ cytokine storm; NF-κB and JAK/STAT activation; MAPK/p38 signaling; SAP–PIX dysfunction; IDO1–kynurenine inflammatory metabolism.	Usually not defined by recurrent tumor mutations; inherited HLH genes (PRF1, UNC13D, STX11/STXBP2, SH2D1A/XIAP) should be evaluated; clonal T/NK disease may coexist.	HLH-2004 criteria; ferritin, sCD25, triglycerides, fibrinogen and NK-cell activity; EBV DNA; EBER-ISH or sorted-cell EBV testing; IFN-γ/IL-10/IL-18 profiling; lineage assessment to exclude CAEBV/ANKL.	Etoposide/dexamethasone-based HLH therapy; rituximab when B-cell EBV burden is relevant; ruxolitinib or IFN-γ/IL-1 blockade; EBV-specific T cells; HSCT for familial, refractory or underlying clonal disease.
Hydroa vacciniforme-like lymphoproliferative disorder (HV-LPD)	Local EBV latency in cutaneous T/NK cells; LMP1/EBNA1-associated NF-κB/JAK-STAT and cytokine loops; UV-associated inflammatory microenvironment.	EBV-positive T/NK clonal or oligoclonal expansion; recurrent driver mutations remain poorly defined; systemic cases may acquire additional genetic abnormalities.	Recurrent photosensitive vesiculopapular lesions and scarring; skin biopsy with EBER+ T/NK infiltrates and cytotoxic markers; EBV DNA; assessment for systemic symptoms and CAEBV overlap.	Photoprotection and supportive care in classic disease; systemic/progressive disease may require immunochemotherapy and HSCT; EBV-specific CTLs, JAK/STAT and checkpoint approaches remain exploratory.
Severe mosquito bite allergy (SMBA)	Mosquito salivary-antigen-driven CD4+ T-cell help; EBV reactivation in NK/T cells; LMP1/EBNA1-mediated NF-κB and anti-apoptotic signaling; cytokine amplification.	EBV-infected NK-cell lymphocytosis; clonality may be detected; no reproducible recurrent driver mutations have been established.	Exaggerated mosquito-bite reaction with necrosis/ulceration plus fever, lymphadenopathy or hepatosplenomegaly; EBV DNA; EBER+ T/NK cells; IgE/NK-cell expansion; evaluation for CAEBV/HV-LPD.	Bite avoidance and anti-inflammatory control for local disease; treat associated CAEBV/HLH when present; HSCT for severe systemic progression; EBV-specific and cytokine-pathway strategies are investigational.

EBV, Epstein–Barr virus; EBER-ISH, EBV-encoded RNA *in situ* hybridization; HSCT, hematopoietic stem cell transplantation; NGS, next-generation sequencing; CTLs, cytotoxic T lymphocytes.

### 4.1 Chronic active EBV infection

CAEBV is a rare but clinically challenging disease, usually associated with persistent EBV infection and expansion of infected T or NK cells, and is characterized by systemic inflammation, organ involvement, and progressive tissue damage. EBV DNA load is one of the most commonly used molecular markers for diagnosing and assessing the prognosis of CAEBV. However, EBV DNA load should not be interpreted as a direct surrogate for malignant transformation or active lytic replication. Its clinical significance depends on the sample type, detection platform, and cellular compartment analyzed. In CAEBV, identifying EBV-infected T- or NK-cell lineages by EBER *in situ* hybridization, flow cytometry-based cell sorting, or lineage-specific molecular assays is particularly important, because whole-blood or plasma EBV DNA levels alone cannot fully distinguish CAEBV from other severe EBV-associated conditions. An elevated EBV DNA copy number in peripheral blood (PB), such as >10².^5^ copies/μg, has been included as an auxiliary diagnostic criterion for CAEBV (130). Elevated viral load often indicates active viral replication and is closely associated with disease activity and prognosis (131, 132). However, it is important to note that although CAEBV patients have significantly higher EBV DNA loads than those with acute infections (e.g., infectious mononucleosis, IM), the difference is not absolute when compared to other severe EBV-related diseases such as EBV-positive lymphomas or hemophagocytic lymphohistiocytosis (EBV-HLH) (133). Therefore, EBV DNA load should be evaluated in conjunction with other indicators, such as tissue EBER *in situ* hybridization, identification of the infected T/NK-cell subtype, clonality assessment, and clinical presentation, to improve the accuracy of CAEBV diagnosis and dynamic monitoring.

From a molecular perspective, the core feature of CAEBV is chronic immune activation driven by persistent EBV infection of T/NK cells, accompanied by sustained expansion of infected lymphocytes and, in some cases, clonal evolution toward overt T/NK-cell malignancy. In CAEBV, NF-κB and JAK-STAT signaling may be activated through a combination of viral gene expression, cytokine stimulation, chronic inflammatory feedback, and host genetic alterations, promoting cell survival, proliferation, and abnormal cytokine secretion (97–100). These signaling mechanisms, which have been described in detail earlier, are particularly persistent and significant in CAEBV, serving as major drivers of disease progression. It is important to interpret the relationship between CAEBV and malignancy with caution. CAEBV is not simply a uniform precursor lesion, but rather a heterogeneous EBV-driven inflammatory and lymphoproliferative disorder. Some patients show clonal expansion of EBV-infected T/NK cells and acquire additional molecular abnormalities, thereby increasing the risk of progression to overt lymphoma or leukemia. Recent studies have found somatic mutations in T/NK cells from some CAEBV patients, with the most common mutations being in genes such as DDX3X, STAT3, and KMT2D (134, 135). These mutations are also observed in other T/NK-cell lymphomas, suggesting that a subset of CAEBV cases may share molecular features with malignant T/NK-cell neoplasms and may have a higher risk of disease progression. Additionally, research has revealed large deletions in the EBV genome of some CAEBV patients, particularly in the BamHI A region miRNA cluster and some lytic phase genes. These deletions may alter the balance between latent and lytic viral programs and influence immune recognition or survival of infected cells, but their causal contribution to CAEBV progression remains to be further clarified (136–138). Therefore, changes in the viral genome structure not only reflect adaptive strategies but may also be closely related to the clonal evolution of host cells. CAEBV should be regarded as a biologically heterogeneous disorder positioned between chronic EBV-driven inflammation and clonal T/NK-cell lymphoproliferation, rather than as an inevitable intermediate step toward malignancy. Risk assessment should integrate EBV burden, infected-cell lineage, clonality, host genetic alterations, viral genomic changes, and the extent of systemic organ involvement.

### 4.2 EBV-positive T/NK cell lymphoma

EBV-positive T/NK cell lymphoma is a highly aggressive neoplastic disease driven by persistent EBV infection, originating from T cells or natural killer (NK) cells. Its representative subtypes mainly include nasal-type NK/T-cell lymphoma (NKTCL) and aggressive NK-cell leukemia (ANKL). Although both are closely related to EBV, they exhibit significant differences in the type of viral latency, immune evasion strategies, and molecular mutation profiles.

#### 4.2.1 Nasal-type NK/T-cell lymphoma

NKTCL is a malignancy originating from peripheral NK cells or cytotoxic T cells in the nasopharynx and upper respiratory tract, and it typically presents with EBV-positive characteristics. Studies indicate that NK cells in the nasopharyngeal mucosa can internalize EBV, and subsequently express CD21 receptors abnormally, providing conditions for viral entry. After EBV infection, several cytokines (e.g., IL-2, IL-9, IL-10, IL-15) promote the abnormal proliferation of infected cells via autocrine and paracrine mechanisms. NKTCL usually presents as EBV latent infection type II, primarily expressing EBNA1, LMP1, and LMP2 proteins (139). In this latent state, LMP1 mimics CD40 signaling and activates the NF-κB pathway, enhancing cell survival. However, studies have questioned the extent of NF-κB activation in NKTCL, noting significant individual variability in its activation (140–142). Although the carcinogenic mechanism of EBV in NKTCL is not yet fully elucidated, it may not directly induce malignant transformation but rather indirectly promote tumorigenesis by enhancing the responsiveness to cytokines like IL-2 (27). Recent studies further suggest that LMP1 induces p53 dysfunction, c-Myc activation, and upregulation of survivin expression, thereby activating multiple pro-proliferative and anti-apoptotic pathways that drive the formation and progression of NKTCL (141). Notably, there is some overlap in the viral gene expression profiles between NKTCL and CAEBV cells, suggesting that they may share common pathogenic foundations, supporting the idea that CAEBV could be a precursor state for NKTCL.

#### 4.2.2 Aggressive NK-cell leukemia

ANKL is an extremely poor-prognosis, rapidly progressing hematologic malignancy, with approximately 90% of cases showing positive Epstein-Barr encoding region (EBER), clearly indicating the involvement of EBV infection in its pathogenesis (143–145). Unlike NKTCL, ANKL primarily presents with EBV latent infection type I, expressing only a few latent proteins such as EBNA1, while LMP1 is often negative. This “low immunogenicity” latent mode may help tumorigenic NK cells evade immune clearance (146, 147). In EBV-infected NK cells, in addition to expressing viral latent genes, they also produce immune-regulatory cytokines like IL-10, further activating themselves and maintaining a highly proliferative state (28). Somatic mutations also play an important role in the pathogenesis of ANKL. Studies have found frequent mutations in genes associated with pathways such as STAT3, JAK2, RAS, and MAPK, which enhance cell proliferation signaling and promote immune evasion (148). Furthermore, approximately one-third of ANKL cases exhibit high expression of PD-L1, providing a molecular basis for immune evasion and offering a potential therapeutic target for immune checkpoint inhibitors (149).In terms of viral molecular profiles, although both NKTCL and ANKL are EBV-positive, their viral expression patterns and host responses differ significantly. In NKTCL, LMP1 is highly expressed and often associated with poor prognosis (150, 151), whereas in ANKL, the characteristic feature is EBNA1 positivity and LMP1 negativity, displaying a “silent infection—high invasiveness” pathological feature. Additionally, EBV-encoded BART miRNAs (e.g., miR-BART6, BART13) further promote tumor cell invasiveness and immune evasion by targeting TP53 and immune-regulatory genes, aggravating the clinical malignancy of ANKL (61).

EBV-positive T/NK cell lymphomas exhibit high molecular heterogeneity across different subtypes. NKTCL tends to be tumorigenic through mechanisms of proliferation and inflammation activation mediated by viral proteins like LMP1, while ANKL relies more on the synergy of reduced viral immunogenicity and accumulated host mutations. The differences in latent infection types, gene expression profiles, and immune evasion strategies suggest the adaptability of EBV to different host cell types, with the viral pathogenic mechanism “reprogramming” itself to suit its survival strategy in different immune cell environments. These characteristics not only provide a basis for studying the disease pathogenesis but also offer molecular foundations for accurate diagnosis and subtype-specific treatment. In the future, interventions targeting latent expression products, PD-L1 blockade, and combined mutation-targeted drugs are expected to improve the therapeutic outcomes for EBV-positive T/NK cell lymphomas.

### 4.3 EBV-associated hemophagocytic lymphohistiocytosis

EBV-associated hemophagocytic lymphohistiocytosis (EBV-HLH) is a type II hemophagocytic syndrome induced by EBV infection, primarily occurring in the context of EBV infection of T cells or NK cells. Its characteristics include intense immune activation, cytokine storm, and multi-organ dysfunction, presenting with an “inflammation-dominant” phenotype in NK/T cell diseases.

#### 4.3.1 Mechanisms of cytokine storm induced by EBV infection

The core mechanism of EBV-HLH pathogenesis is uncontrolled infectious immune activation (immunopathology). During EBV infection of host cells, particularly CD8+ T cells or NK cells, several viral and host factors contribute to the amplification and maintenance of the cytokine storm. First, EBV infection can lead to a deficiency in signaling lymphocytic activation molecule-associated protein (SAP), which impairs its interaction with guanine nucleotide exchange factor PIX and signal transduction (152). After the SAP-PIX pathway is blocked, T cells are unable to complete cytotoxic actions and instead over-secrete Th1-type cytokines (such as interferon-gamma, IFN-γ), resulting in sustained inflammatory stimulation. Second, the EBV latent membrane protein LMP1 can activate the NF-κB signaling pathway, leading to the upregulation of pro-inflammatory cytokines such as IL-6, TNF-α, and IL-18 (153). LMP1 can also induce epidermal growth factor receptor (EGFR) expression, enhancing T cell activation and growth response, further exacerbating the cytokine storm. The combined effect of these aberrant immune signals leads to widespread activation of immune cells (such as macrophages and CD8+ T cells), extensive phagocytosis of hematopoietic cells, and the release of pro-inflammatory mediators, ultimately resulting in the classic pathological features of HLH, including fever, hepatosplenomegaly, cytopenia, and hyperferritinemia, as well as other systemic manifestations.

#### 4.3.2 Correlation between viral load and disease severity

The EBV DNA viral load in peripheral blood is a crucial indicator for evaluating the severity and dynamics of EBV-HLH. Most studies indicate that an elevated viral load correlates with the degree of immune activation, organ dysfunction, and prognosis. In EBV-HLH patients, significantly increased viral loads often suggest active viral replication within T/NK cells, which is associated with high levels of IFN-γ, sCD25, IL-18, and other cytokines. Persistent high viral loads usually indicate insufficient immune clearance of the virus, which is closely linked to disease refractoriness, recurrence, and an increased risk of mortality (154). Therefore, viral load is not only an important diagnostic and disease stratification tool but also an irreplaceable dynamic marker for monitoring treatment responses, such as antiviral therapy, chemotherapy, and post-stem cell transplant relapse risks.

#### 4.3.3 Distinct mechanism of EBV-HLH compared to other tumor diseases

As an “inflammatory storm-type” T/NK cell proliferative disease induced by EBV infection, EBV-HLH differs in pathogenesis from tumor diseases like NKTCL and ANKL. Its primary feature is that it involves immune activation failure rather than clonal malignant transformation. The virus mainly expresses latent proteins, activating the NF-κB and Th1 cytokine axes. Viral load and cytokine levels are highly correlated, making them key indicators for disease monitoring. Some cases may transition to ANKL or CAEBV, suggesting a bridging role within the EBV lymphoproliferative disease (LPD) spectrum. Therefore, EBV-HLH is not only a result of EBV-induced immune regulation failure but may also represent an intermediate stage in the malignant progression of some CAEBV cases. Future research should further explore how virus-host interactions influence the initiation, maintenance, and resolution of HLH, particularly in the context of immune therapies (such as IFN-γ antagonists, JAK inhibitors) and combined antiviral strategies, offering new therapeutic insights.

### 4.4 Other EBV-associated T/NK cell lymphoproliferative diseases

In addition to the major diseases such as CAEBV, NKTCL, ANKL, and EBV-HLH, EBV can also trigger several T/NK cell-related proliferative disorders with characteristics of skin and allergic reactions. Although these diseases are relatively rare clinically, their pathogenesis is closely related to viral infection, immune responses, and abnormal cell activation, further enriching the clinical and biological features of the EBV-associated T/NK cell disease spectrum.

#### 4.4.1 Hydroa vacciniforme-like lymphoproliferative disorder

HV-LPD (Hydroa vacciniforme-like lymphoproliferative disorder) is a rare but clinically significant EBV-positive T/NK cell-related disease, primarily characterized by recurrent vesicular skin lesions, often occurring in children and adolescents. The pathological mechanism involves the persistent proliferation of EBV-infected T cells or NK cells in skin tissues and focal inflammatory responses (155). Based on clinical presentation and prognosis, HV-LPD can be divided into two subtypes (156):

#### 4.4.2 Classic HV-LPD

Characterized by symmetric vesicular rashes on the face and exposed areas, with a relatively stable course and self-limiting nature. Systemic symptoms or organ involvement are rare.

#### 4.4.3 Systemic HV-LPD

Besides aggravated skin lesions, it is often associated with fever, hepatosplenomegaly, lymphadenopathy, and cytopenia. This subtype frequently indicates disease progression or malignant transformation, and some patients may eventually develop systemic T cell lymphoma (3, 156–160).

The role of EBV in HV-LPD remains incompletely understood, but studies suggest that latent viral proteins, such as LMP1 and EBNA1, can be expressed in lesional tissues, indicating that persistent viral infection and immune microenvironment remodeling play a key role in its pathogenesis.

#### 4.4.4 Severe mosquito bite allergy

SMBA (Severe mosquito bite allergy) is another skin allergic reaction-related disease closely associated with EBV infection. It is characterized by an abnormal immune overreaction to mosquito bites, often accompanied by edema, necrosis, fever, hepatosplenomegaly, and systemic inflammatory manifestations. Recent studies have found that CD4+ T cells in SMBA patients show a significant immune response to mosquito salivary gland extract (SGE), suggesting that the disease follows a tripartite mechanism involving exogenous antigen induction, viral reactivation, and immune amplification (161). SGE antigen stimulation can induce EBV reactivation, particularly in latent T/NK cells. The EBV-infected T/NK cells are subsequently reactivated at the site of mosquito bites, with elevated expression of viral proteins such as LMP1 and EBNA1, which block apoptosis and enhance cell survival. The persistent expression of EBV oncogenes promotes local inflammation, tissue damage, and immune evasion, potentially leading to disease progression and even evolution into T cell lymphoma (162).

Since SMBA is often comorbid with CAEBV or HV-LPD, some studies consider it an immune activation subtype of EBV-associated skin T cell diseases, highlighting its special role in the EBV disease spectrum. Both HV-LPD and SMBA, although with relatively low clinical incidence, are closely related to persistent EBV infection of T/NK cells, viral activation, and immune dysregulation. They exemplify how EBV can establish latent infection in non-hematopoietic tissues and induce local or systemic immune disorders. These conditions:(1)Represent typical examples of EBV infection presenting with a skin preference within the EBV infection spectrum.(2)May serve as potential precursor states or comorbid subtypes for systemic diseases like CAEBV and NKTCL.(3)Show high overlap in histological features, viral protein expression profiles, and immune checkpoint expression with other systemic EBV-related diseases. Therefore, further research on HV-LPD and SMBA not only helps to understand the adaptation and evolutionary strategies of EBV in different tissue microenvironments but also provides new insights for exploring immune interventions and virus-specific therapies.

## 5 Discussion and future directions

Epstein-Barr virus (EBV)-associated NK/T cell lymphoproliferative diseases continue to face numerous challenges in terms of pathogenesis, diagnosis, and treatment strategies. With the rapid development of virology, immunology, molecular biology, and artificial intelligence, future research should not only identify new biomarkers and therapeutic targets, but also clarify which EBV-related mechanisms are directly supported in T/NK-cell diseases and which are extrapolated from B-cell or epithelial tumor models. Particular attention should be paid to the biological boundary between EBV-driven inflammation, clonal lymphoproliferation, and overt malignancy, especially in CAEBV and EBV-HLH.

### 5.1 Interaction between virus and host genome

The interaction between the virus and the host genome is an important area of research in EBV-associated T/NK-cell lymphoproliferative disorders, but current evidence remains more limited than that available for EBV-positive B-cell or epithelial tumors. EBV’s genome can affect the gene expression and function of host cells through various mechanisms. Evidence from non-T/NK EBV-associated tumors provides useful mechanistic references. For example, in EBV-associated gastric cancer (EBVaGC), EBV infection upregulates the HUSH complex (particularly TASOR) and increases H3K9me3 modifications, selectively suppressing younger L1 and FL-L1 retrotransposons with reverse transcriptase activity (163). Additionally, CRISPR gene editing technology provides a powerful tool to study the interactions between EBV and the host genome. Through CRISPR/Cas9 screening, scientists have discovered that host miR-142 plays a key role in the transition from EBV latency to lytic replication. Deletion of miR-142 significantly increased the expression of EBV lytic genes and enhanced Erk/MAPK signaling after B cell receptor (BCR) stimulation. miR-142 inhibits the transition of EBV from latent to lytic phase by directly targeting the SOS1/Ras/Raf/Mek/Erk signaling axis, thus limiting EBV reactivation. Furthermore, small molecule inhibitors targeting miR-142-regulated signaling pathways have been shown to suppress EBV lytic reactivation, offering potential therapeutic strategies for related diseases (164). However, because this mechanism was defined in the context of B-cell receptor-related signaling, its relevance to T/NK-cell lymphoproliferative disorders should be considered indirect. Future studies using EBV-positive T/NK-cell models, single-cell multi-omics, and lineage-specific functional validation are needed to determine whether comparable virus-host regulatory circuits operate in T/NK-cell diseases.

### 5.2 New molecular biomarkers and therapeutic targets

The development of diagnostic biomarkers based on molecular mechanisms and targeted therapeutic strategies is an important direction for future research in EBV-associated NK/T-cell lymphoproliferative disorders. Targeting EBV-driven IDO1-NAD metabolism has made significant progress as a potential therapeutic strategy in EBV-infected B-cell models. EBV-infected B cells activate IDO1 to promote *de novo* NAD^+^ synthesis, supporting cell proliferation and transformation. Inhibiting IDO1 activity or NAD^+^ synthesis can significantly inhibit the transformation of EBV-infected B cells, providing a potential metabolic target for EBV-driven lymphoproliferation. Moreover, EBNA2^+^IDO1^+^ B cells and high IDO1 activity markers, such as the kynurenine/tryptophan ratio, may serve as potential biomarkers for predicting the development of EBV-related diseases such as post-transplant lymphoproliferative disorder (PTLD) (165). However, these findings are mainly derived from B-cell systems, and their applicability to EBV-positive T/NK-cell diseases still requires disease-specific validation.

At the same time, EBV-encoded miRNAs exhibit unique expression patterns in various EBV-related diseases, with potential for use as disease prediction, diagnosis, prognosis, and therapeutic targets (61). For example, in infectious mononucleosis, the dynamic expression of specific EBV-miRNAs, such as BART16, can be used as biomarkers for monitoring disease progression (166); in EBV-associated hemophagocytic lymphohistiocytosis (EBV-HLH), high expression of miRNAs such as BART3-3p is associated with the cytokine storm, potentially serving as therapeutic targets (167); in chronic active EBV infection, miRNAs such as BART2-5p and BART13 can be used as markers to assess disease activity and prognosis (168); in EBV-related tumors, overexpression of miRNAs such as BART1-5p and BART13 can be used to differentiate malignant diseases from benign lesions and guide diagnosis and treatment (169). These findings suggest that EBV miRNAs may provide complementary information to EBV DNA load and conventional EBV detection, especially in diseases with limited or heterogeneous viral protein expression. Nevertheless, their diagnostic and prognostic value in CAEBV, EBV-HLH, NKTCL, and ANKL should be evaluated separately because these entities differ in viral gene expression, host-cell lineage, and clinical behavior.

In terms of treatment strategies, targeted therapy against EBV-related signaling pathways has become a research hotspot. Proteasome inhibitor bortezomib induces apoptosis in EBV-positive lymphoma cells by inhibiting the NF-κB pathway (170); PI3K-Akt-mTOR pathway inhibitors, such as MK2206 and rapamycin, have shown efficacy in EBV-associated lymphomas (171–173); PD-1/PD-L1 immune checkpoint inhibitors, such as pembrolizumab, have shown significant effects in treating NK/T-cell lymphoma, with response rates ranging from 57.1% to 100% (149, 174). Combination therapy strategies also show potential. For instance, the combination of mTOR inhibitors and PI3Kδ inhibitors enhances the treatment effect on EBV-positive B-cell lymphoma (175); the combination of histone deacetylase inhibitors, such as valproic acid, and proteasome inhibitors, such as bortezomib, has been shown to have anti-tumor effects on EBV-associated lymphomas (176, 177). These findings provide useful therapeutic references, but their direct relevance to EBV-positive T/NK-cell lymphoproliferative disorders should be interpreted cautiously because T/NK-cell diseases differ from B-cell lymphomas in host-cell lineage, viral latency pattern, immune microenvironment, and clonal evolution.

In cellular immunotherapy, EBV-specific T-cell immunotherapy has achieved long-term remission in the prevention and treatment of post-transplant EBV-related lymphoma, and cytotoxic T cells targeting EBV latent membrane proteins, such as LMP1 and LMP2A, also show good prospects in the treatment of EBV-related lymphoma (178). For EBV-positive T/NK-cell disorders, the efficacy of this strategy may depend on the actual expression of target antigens, the degree of viral protein heterogeneity, and the immune status of the patient. In recent years, emerging therapeutic directions for EBV-related cancers have been continuously expanded. Strategies targeting free-floating EBV genomes show potential; for example, small molecule inhibitors targeting the EBNA1 protein can eliminate EBV genomes in cancer cells (179, 180). Additionally, lysis-inducing therapies utilize the presence of free EBV genomes to express viral protein kinases, achieving therapeutic goals (181). However, these approaches face several challenges, including heterogeneous viral antigen expression, uncertain activity in T/NK-cell disease models, and the difficulty of distinguishing antiviral effects from anti-tumor effects. Autologous dendritic cell vaccines based on EBV antigens, such as KSD-101, have shown promise in activating anti-tumor immune responses. Antiviral drugs, such as tenofovir alafenamide, may reduce viral load by inhibiting EBV DNA polymerase, providing new avenues for selected EBV-related diseases. These emerging therapies bring new hope for EBV-related cancers, but their clinical translation in EBV-associated T/NK-cell lymphoproliferative disorders still requires large-scale studies to validate their safety and general applicability.

### 5.3 Personalized medicine

Biomarker-driven precision diagnostics and treatments have shown great promise in the management of EBV-associated T/NK-cell lymphoproliferative disorders, but they also face disease-specific challenges. Biomarkers, as measurable indicators of biological processes or states, can provide crucial information for clinical decision-making and enable personalized cancer care. For EBV-positive T/NK-cell diseases, precision medicine should integrate EBV DNA load, tissue EBER *in situ* hybridization, high-sensitivity viral detection, infected-cell lineage identification, clonality assessment, host genomic alterations, immune checkpoint status, and clinical inflammatory activity. Traditional biomarker testing typically relies on a single data modality, such as genomics, histology, or radiology, which may not fully capture the complexity and heterogeneity of these diseases. Additionally, most conventional biomarker testing requires invasive procedures, which limits its clinical applicability.

To overcome these limitations, artificial intelligence (AI) technologies, by integrating multimodal data, including clinical, radiological, histological, genomic, and laboratory data, may offer more accurate clinical predictions and uncover new non-invasive biomarkers and treatment-related indicators. For example, AI models can identify associations between genomic mutations and tissue morphology, or establish links between radiomic features and histological subtypes, thereby assisting in early diagnosis, risk stratification, and treatment selection (182). In EBV-associated T/NK-cell diseases, such integration may help distinguish chronic EBV-driven inflammation, clonal lymphoproliferation, and overt malignancy, especially in clinically heterogeneous conditions such as CAEBV and EBV-HLH. However, AI-based precision medicine strategies must be built on well-annotated disease-specific cohorts; otherwise, heterogeneous EBV detection methods, variable tumor-cell content, and misclassification of EBV-low or EBV-negative cases may introduce bias.

In EBV-related diseases, the integration of whole-genome sequencing and AI-based precision medicine strategies has demonstrated significant potential. However, challenges remain, such as high technical costs, data privacy and security issues, and a lack of clinical standardization. Future efforts should focus on strengthening interdisciplinary collaboration, balancing technological advancement with ethical oversight, and establishing large-scale data service platforms. Improving data governance frameworks will ensure data security and compliance, which is essential for the broad implementation of personalized treatment approaches. For EBV-associated T/NK-cell lymphoproliferative disorders, standardized EBV testing, harmonized reporting of viral load compartments, lineage-specific identification of infected cells, and prospective validation of biomarkers will be essential for improving diagnosis, risk stratification, and individualized treatment selection.

## 6 Conclusion

EBV is a widely prevalent human γ-herpesvirus that can persist in the host and influence immune regulation, cell survival, proliferation, and tumorigenesis through multiple viral and host mechanisms. This review summarizes the molecular basis of EBV-associated NK/T-cell lymphoproliferative disorders, including viral protein-mediated activation of NF-κB, PI3K-AKT, and JAK-STAT signaling pathways, viral miRNA-mediated regulation of apoptosis and immune responses, and immune evasion through checkpoint activation and impaired antigen presentation. However, the biological effects of EBV in T/NK cells should not be interpreted as a simple extension of B-cell or epithelial models, because EBV-positive T/NK-cell diseases show distinct features in viral gene expression, host-cell lineage, immune microenvironment, and clonal evolution.

EBV-associated T/NK-cell lymphoproliferative disorders, including CAEBV, NKTCL, ANKL, and EBV-HLH, represent a clinically and biologically heterogeneous disease spectrum. CAEBV is better regarded as a chronic EBV-driven inflammatory and lymphoproliferative disorder with variable risk of clonal progression, whereas EBV-HLH is primarily characterized by excessive immune activation and cytokine storm. These distinctions highlight the need to integrate viral load monitoring, tissue-based EBV detection, infected-cell lineage analysis, clonality assessment, and host molecular features in disease classification and risk stratification. Although EBV-encoded miRNAs, EBV-related signaling pathways, PD-1/PD-L1 blockade, and EBV-specific cellular immunotherapy provide promising diagnostic and therapeutic directions, standardized treatment strategies for EBV-associated T/NK-cell lymphoproliferative disorders remain insufficient. Future studies should further clarify the lineage-specific mechanisms of EBV infection in T/NK cells, validate reliable biomarkers in disease-specific cohorts, and optimize multimodal diagnostic approaches, ultimately improving early recognition, individualized intervention, and patient outcomes.
